# Prevalence of and factors associated with female sexual dysfunction among women using hormonal and non-hormonal contraception at the AGA Khan University Hospital Nairobi

**DOI:** 10.4102/phcfm.v11i1.1955

**Published:** 2019-10-16

**Authors:** Momin R. Butt, Valentino Lema, Abraham Mukaindo, Gulnaz Mohamoud, Jacob Shabani

**Affiliations:** 1 Department of Obstetrics and Gynaecology, Aga Khan University Hospital, Nairobi, Kenya

**Keywords:** prevalence, female sexual dysfunction, contraception, hormonal, non-hormonal

## Abstract

**Background:**

Female sexual function (FSD) is a complex phenomenon. It integrates all body systems and is influenced by a variety of factors. Contraceptives have shown to have variable effects on FSD. In Kenya, the majority of women use hormonal contraception with high rates of discontinuation of use, attributed to related side effects such as weight loss and loss of libido.

**Aim:**

To determine the prevalence of and the factors affecting FSD among women using contraception in our setting.

**Setting:**

The study was carried out at the Aga Khan University Hospital, Nairobi, at various clinical sites.

**Methods:**

A cross-sectional study was conducted. Consecutive sampling of women of reproductive age using either hormonal or non-hormonal contraception was conducted. Two questionnaires were completed after obtaining informed consent. Independent associations of factors with the outcome variables were assessed using the chi-square test of association, and variables with a *p* < 0.25 were used in the multivariate analysis. Factors associated with FSD were determined using binary logistic regression.

**Results:**

A total of 566 participants were included. The prevalence of FSD among those using hormonal and those using non-hormonal contraception was 51.5% and 29.6%, respectively (*p* < 0.0001). We found that the factors associated with FSD were presence of chronic illness and use of chronic medication, being self-employed or unemployed, alcohol intake and history of miscarriage(s).

**Conclusion:**

There was a high prevalence of and a strong association between hormonal contraception and FSD. More studies on this topic in different settings are recommended to investigate the effect of each type of hormonal method on FSD.

## Introduction

Sexual function is an important component of sexual health that determines the general well-being of a person.^1^ Female sexual dysfunction (FSD), which is the decrease in sexual desire or arousal, or the presence of dyspareunia and difficulty/inability to achieve orgasm, can cause personal distress.^1^ Female sexual dysfunction is a common problem globally, and the factors associated with it have been highlighted in the literature and broadly divided into five main groups. These include biological factors such as hormonal status that can be influenced by the use of hormonal contraception; demographic factors such as age and education levels; psychological factors, including mental health conditions, such as anxiety and depression; sociocultural factors such as religion and traditional customs; and finally the pathophysiological factors that are complications associated with chronic conditions such as diabetes mellitus and rheumatoid arthritis. These factors have been illustrated in the biopsychosocial model of FSD. All the above-described factors are far from having a predictable effect on sexual function and have been shown to have variable effects in different studies. They are defined as the association of demographic factors such as increasing age, increasing parity and lower levels of education, which have been shown to have a significant negative effect on sexual function.^2^ Other studies draw completely opposite conclusions, so no current consensus exists on predictability of the effect of most of the above factors.

The current female sexual response model is a circular one, which was elaborated by Basson in the year 2000 and has evolved from two previous linear models: the first described by Masters and Johnson in 1966 and the other that had eliminated one of the previously described stages by Kaplan et al. in 1979. The non-linear model acknowledges and addresses the effect of the other factors, such as relationship status and emotional receptivity, on the outcome of sexual response and is, therefore, generally more acceptable.^3^

Contraception is one such factor, the biochemical effects of which have a clear association with sexual function, such as reduced vaginal lubrication, leading to dyspareunia. The current contraceptive prevalence rate in Kenya is 58%, with the majority of women using hormonal methods. Significant proportions (31%) of the users discontinue use within 1 year of starting because of side effects of the contraceptive methods.^4^

Sexuality is a taboo subject in many societies, and women and physicians do not routinely discuss effects of contraception on sexual health. It was therefore important to examine the effect of hormonal contraception on FSD to have contextually relevant information for women seeking to be on or already on contraception because a high number of women are already using hormonal contraception methods.

This study aimed to compare the prevalence of sexual dysfunction in women attending the Aga Khan University Hospital, Nairobi (AKUHN) clinics using hormonal contraception to those using non-hormonal contraception, to determine and examine the other factors associated with FSD.

## Research methods and design

### Study design

This was an analytical, cross-sectional survey.

### Study setting

The study was carried out at the Aga Khan University Hospital, which is a tertiary care referral hospital that generally serves a wide variety of individuals with different social and demographic characteristics. The clinics identified to be used for the study were the family planning clinic, family medicine clinic and gynaecology outpatient clinic.

### Study population and sampling strategy

The cohort studied were women of reproductive age (18–49 years) who were using any form of contraception. To be recruited, participants had to be aged 18 years or above to give informed consent. Consecutive sampling technique was used to recruit participants from the clinics until the required sample size was achieved.

Women attending the above-named clinics, who were in the specified age category, were approached by the triage nurse. The eligibility of the subjects was confirmed using a set of three questions about their age, whether they spoke English and if they were using any contraceptive. The eligible women who agreed to participate in the study were given two questionnaires: the demographic questionnaire and the Female Sexual Function Index (FSFI) to be filled in a separate private room. The participants were asked to drop them in a sealed box at the triage station after they had filled them.

#### Inclusion criteria

Women aged between 18 and 49 years who attended the AKUHN clinics, and who could read and write in English were included in the study. This was mainly because the study questionnaire had not been validated in Kiswahili or any other local language from our set-up. The participants also had to be sexually active and in a heterosexual relationship for the past 4 weeks prior to participating in the study. The participants had to be using any type of contraception, particularly only one type of contraception for the past 6 months.

#### Exclusion criteria

Women who had a known psychiatric illness and history of sexual dysfunction were excluded from the study. Also, women who had undergone pelvic floor surgery, had chronic pelvic pain syndromes or a history of any gynaecological malignancy were excluded, as these conditions are known to have a negative influence on sexual function. Women who had switched from one type of contraception to another in the past 6 months were also excluded as this is the duration beyond which, according to the DSM-5, a diagnosis of sexual dysfunction can be made from persistence of particular symptoms.

## Data collection

### Sample size calculation

The formula used for sample size determination was that for comparison of proportions between two groups:

$$n=\frac{\left[p_{1}(1-p_{1})+p_{2}(1-p_{2})\right]}{{\left(p_{1}-p_{2}\right)}^{2}}\times c_{\text{p}.\text{power}}$$ [Eqn 1]

where *n* is the sample size, *P*_1_ is the prevalence of sexual dysfunction in proportion one and *P*_2_ is the prevalence of sexual dysfunction in proportion two. The power of the study was set at 80%. The value of *α* was set at 0.05 (95%). The *C*_p.power_ was 7.9. Using the 2017 study by Oindi et al. (an unpublished study conducted at AKUHN), the prevalence they obtained from their study was used for calculating sample size, where *P*_1_ was the prevalence of sexual dysfunction in the group of women in the study who were using non-hormonal contraception. We aimed to detect at least a 10% difference (*P*_1_ = 26.9%)^5^ in proportion with FSD in this group. A 10% difference in prevalence of FSD between the two groups was thought to be clinically significant. *P*_2_ was the prevalence of sexual dysfunction in the cohort of women attending AKUHN clinics using hormonal contraception, reported as 36.9%. The calculations using the above formula for comparison of proportions gave a sample size of 283 women in each arm. This was then multiplied by two to get a total sample size of 566 women.

A two-part questionnaire was used to collect the data. The first part was about sociodemographic information and the second part entailed sexual function in women.

The sociodemographic questionnaire collected information on the age of the subject; the age of the partner; parity; race; marital status; religion; alcohol intake; smoking status; employment status; presence of chronic illnesses, for example, hypertension, diabetes mellitus and rheumatoid arthritis; if they were currently on any medication for the aforementioned conditions; and the type of contraception that was currently being used (hormonal or non-hormonal).

The FSFI questionnaire was used to collect information on the outcome variable. This is a 19-item questionnaire that was developed as a brief, multidimensional self-report tool to assess the important aspects of sexual function in women. It is a generally well-accepted tool and has been used in several studies across the world. It is easier to administer than most in terms of simplicity. It has also been tested for validity and inter-rater cross-reliability. The features examined in the desire domain include frequency of desiring intercourse, level of desire of intercourse, satisfaction and confidence with performing intercourse. The arousal domain included frequency of successful intercourse and difficulty in getting aroused for intercourse. The lubrication domain included frequency of attaining and difficulty in maintaining lubrication. The pain domain assesses frequency of pain during vaginal penetration, frequency following vaginal penetration and level of pain during and following vaginal penetration. The satisfaction domain mainly assessed factors like satisfaction with the amount of closeness with the sexual partner, satisfaction with the sexual relationship and, in general, satisfaction with one’s sexual life.^6^

### Data analysis

At the end of the day, the questionnaires were checked for completion by the triage nurse who then filed the questionnaires and stored them in a locked cupboard for collection. The data from the forms were entered into a Microsoft Excel sheet in a password-protected computer and later analysed using SPSS (Statistical Package for the Social Sciences) version 22.

Continuous variables were summarised using means or median with their corresponding measures of variability (standard deviation [s.d.] or interquartile range [IQR]), while categorical data were summarised using frequency counts with corresponding percentages. Prevalence of FSD was calculated as a proportion of the women with FSD in the population of all women interviewed. This was stratified by the type of contraceptive (hormonal and non-hormonal) used, and the two proportions were compared using two sample tests of independent populations.

The FSFI scores were calculated using the responses to the questions from each of the domains. All questions had a score range of 1 to 5, with two questions for desire domain; four questions each for arousal and vaginal lubrication, orgasm; and three questions each for pain and sexual satisfaction. The total score was calculated by adding the scores in a particular domain, and then multiplying it by a set coefficient for each domain. The set coefficients were 0.3 for sexual arousal and vaginal lubrication, 0.6 for desire and 0.4 for orgasm, sexual pain and sexual satisfaction. The final FSFI score was then calculated by adding all the scores for each domain and this ranged between 2 and 36.

Female sexual dysfunction was measured on a categorical binary scale where any woman with a total score of less than 26.55 is considered to have sexual dysfunction and higher scores are indicative of better sexual function (without FSD). This tool was developed and validated by Rosen, and its reliability was calculated using Cronbach’s alpha for internal reliability and multivariate analysis of variance, dysfunction diagnosis *x* FSFI domain with Bonferroni-corrected *post hoc* comparisons to be 0.82.

Independent associations of the explanatory factors with the outcome variable were assessed using chi-square test of association, and the variables with a *p* < 0.25 were used in the multivariate analysis. Factors associated with FSD were determined using binary logistic regression adjusting for the influence of the variables on each other or any possible confounding factor.

### Ethical considerations

The study commenced after ethical approval from the Aga Khan University ethics review committee’s consideration. Informed consent was obtained prior to recruiting participants into the study, and it was made clear that participants will be free of coercion, inducement or intimidation. Participants in the study did not receive any reward. However, declining to participate in the study did not interfere with the patients’ intention of attending the respective clinic.

## Results

A total number of 596 participants were recruited from three clinics: gynaecology outpatient clinic, family medicine clinic and family planning clinic over the study duration of February to April 2018. They submitted completed questionnaires of which 30 (5%) were excluded because of incomplete data. Data from 566 participants were included in the analysis after checking for data completeness and confirmation of informed consent and eligibility criteria. The mean age of the participants was 32 years with an age range of 18–49 years, whereas the mean age of their partners was not much higher at 36 years. Most of the participants were married (67%), and the majority had attained tertiary education (82%). A large majority (98%) were African. Sixty-two per cent had salaried employment. Only 13% had a chronic illness and 10% were on chronic medication. Most (67%) of the women were multiparous and 9% of the participants had had at least one miscarriage. Christians comprised the majority (97%) and 30% of women used alcohol of which 9% used it within recommended limits of two units a day and 21% used alcohol in excess of the recommended amount for women. A minority (6%) of them were active smokers. This has been elaborated in Table 1.

**TABLE 1:** Sociodemographic characteristics of the study participants.

Social and/or demographic characteristics domain	*n*	%
**All study participants**	566	100.0
**Age of subjects (years)**		
Mean age	32.3	-
**Age of partners (years)**		
Mean age	36	-
**Level of education**		
Primary education	9	1.6
Secondary education	93	16.4
Tertiary education	464	82.0
**Relationship status**		
Single	116	20.5
Married	381	67.3
Separated	9	1.6
Divorced	5	0.9
Other†	55	9.7
**Race**		
African	557	98.0
Asian	6	1.1
Other‡	3	0.5
**Employment status**		
Salaried employment	355	62.7
Self-employed	137	24.2
Unemployed	74	13.1
**Chronic illness**		
Present	73	12.9
Absent	493	87.1
**Use of chronic medication**		
Present	55	9.7
Absent	511	90.3
**Previous pregnancies**		
Nulliparous	188	33.2
Multiparous	378	66.8
**Miscarriages**		
Yes	53	9.4
No	513	90.6
**Religion**		
Christian	549	97
Islam	10	1.8
None	6	1.1
Other§	1	0.2
**Alcohol use**		
Yes	170	30.0
No	396	70.0
Within recommended limits	48	8.5
Excess of recommended limits	122	22.0
**Smoking status**		
Yes	33	5.8
No	533	94.2
**Use of hormonal contraception**		
Yes	235	41.5
No	331	58.5

† , Others in ‘relationship status’ included ‘engaged’, ‘in a relationship’, ‘dating’, ‘good friends’, ‘couple’, ‘co-habiting’.

‡ , Others in ‘race’ included ‘Arab’, ‘African-American’, ‘mixed-race people’.

§ , Other in ‘religion’ included ‘atheist’.

### Contraceptive use and female sexual dysfunction

**FIGURE 1:**
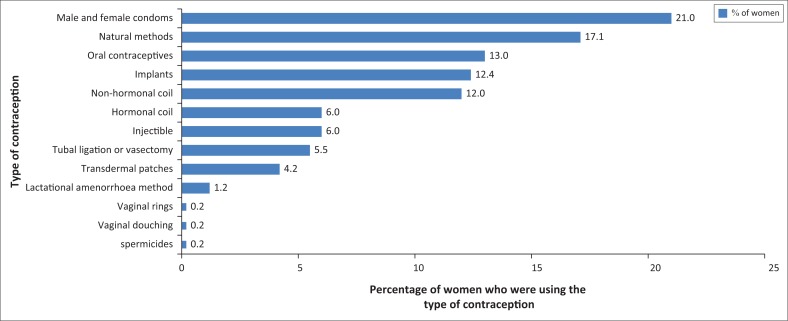
Frequency of use of contraceptive methods by the study participants.

Majority of the participants were using non-hormonal contraception (58.5%), mainly male and female condoms (21%), natural methods (17%) and the intrauterine device (Copper T) (12.%). The rest (41.5%) were using hormonal methods, including oral contraceptive pills (13.7%), implants (12.4%) and levonorgestrel-releasing intrauterine system (6.9%), as shown in Figure 1.

We used the defined cut-off score of 26.55 points to determine the participants who had sexual dysfunction. The overall prevalence of FSD was 38.7% (219 of the 566 participants) in this study population. The hormonal contraceptive users had a prevalence rate of 51.5%, while the non-hormonal contraceptive users had a prevalence rate of 29.6%, which was a significant difference. The prevalence of FSD for each of the contraception types is presented in Table 2. The prevalence was highest in implant users (63%), followed by those on oral contraceptive pill (57%).

**TABLE 2:** Contraceptive use and prevalence of female sexual dysfunction.

Variable	*n* with FSD	Total eligible	Prevalence of FSD (%)
**All study participants**	219	566	38.7
**Participants on hormonal contraceptives**	121	235	51.5
Implants	44	70	62.9
Oral contraceptive pill	42	74	56.8
Injectable	16	34	47.1
Hormonal coil	15	39	38.5
Transdermal patches	7	24	29.2
Vaginal rings	1	1	100.0
**Participants on non-hormonal contraceptives**	98	331	29.6
Male and female condoms	42	119	35.3
Natural methods	42	97	35.3
Non-hormonal coil	19	68	27.9
Tubal ligation	8	31	25.8
Vaginal douching	0	1	0.0
Spermicides	0	1	0.0

FSD, female sexual dysfunction.

We examined the association between the various social and demographic factors among all women in the study group (Table 3).

**TABLE 3:** Prevalence of female sexual dysfunction across different categories of sociodemographic factors.

Variables	Frequency/mean	Percent/s.d.	FSD				*p*
			Absence (*n* = 347)		Present (*n* = 219)		
			*n*	%	*n*	%
**Age of participants (mean)**	32.3	7.1	32.5	7.0	32.0	7.4	0.454
**Age of partner (mean)**	36	7.8	36.2	7.8	35.7	8.0	0.502
**Marital status (frequency)**							
Single	116	20.5	67	57.8	49	42.2	0.296
Married	381	67.3	244	64.0	137	36.0	
Separated	9	1.6	4	44.4	5	55.6	
Divorced	5	0.9	2	40.0	3	60.0	
Other	55	9.7	30	54.6	25	45.4	
**Level of education**							0.924
Primary	9	1.6	6	66.7	3	33.3	
Secondary	93	16.4	56	60.2	37	39.8
Tertiary	464	82.0	285	61.4	179	38.6
**Race**							0.835
African	557	98.4	342	61.4	215	38.6	
Asian	6	1.1	3	50.0	3	50.0
Others	3	0.5	2	66.7	1	33.3
**Employment status**							0.239
Salaried employment	355	62.7	227	63.9	128	36.1	
Self-employment	137	24.2	77	56.2	60	43.8
Unemployed	74	13.1	43	58.1	31	41.9
**Use of hormonal method**							< 0.0001
Yes	235	41.5	114	48.5	121	51.5	
No	331	58.5	233	70.4	98	29.6
**Presence of chronic illness**							0.062
Yes	73	12.9	52	71.2	21	28.8	
No	493	87.1	295	59.8	198	40.2
**Use of chronic medication**							0.067
Yes	55	9.7	40	72.7	15	27.3	
No	511	90.3	307	60.1	204	39.9
**Religion**							0.791
Christian	549	97	335	61.0	214	39.0	
Muslim	10	1.8	7	70.0	3	30.0
None	6	1.1	4	66.7	2	33.3
Other	1	0.2	1	100.0	0	0.0
**Alcohol intake**							0.085
Yes	170	30.2	114	66.7	57	33.3	
No	395	69.8	233	59.0	162	41.0
**Smoking status**							0.932
Yes	33	5.8	20	60.6	13	39.4	
No	533	94.2	327	61.4	206	38.6
**Alcohol use**							0.100
Within limits	48	8.5	28	58.3	20	41.7	
None	396	70.0	234	59.1	162	40.9
Excessive	122	21.6	85	69.7	37	30.3
**Parity**							0.818
Nulliparous	188	33.2	114	60.6	74	36.4	
Multiparous	378	66.8	233	61.6	145	38.4
**Miscarriage**							0.054
No	513	90.6	308	60.0	205	40.0	
Yes	53	9.4	39	73.6	14	26.4

FSD, female sexual dysfunction.

The sociodemographic factors found to be significant (variables with a *p* < 0.25) were used in the multivariate analysis, described as Model I in Table 4. The factors associated were then assessed using binary logistic regression adjusting for marital status, level of education and age of self, as described in Model II in Table 4. These factors were assessed even though they did not meet the cut-off significance value, as they are possible confounding factors and because other studies had shown their significance in association with FSD. There was collinearity between alcohol intake and alcohol use; therefore, alcohol use was fit in the multivariate model instead of alcohol intake. We grouped marital status into single, married and others (merged separated, divorced and other), and religion was categorised as Christians and others (Islam, no religion, other religions) in the regression analysis.

**TABLE 4:** Parameter estimates of the factors associated with female sexual dysfunction.

Variables	Model I			Model II		
	OR	95% CI	*p*	OR	95% CI	*p*
**Marital status**						
Single	-	-	-	-	-	-
Married	0.768	0.503–1.173	0.221	0.554	0.333–0.922	0.023
Other†	1.253	0.688–2.282	0.460	1.235	0.658–2.320	0.511
**Level of education**						
Primary	-	-	-	-	-	-
Secondary	1.321	0.311–5.615	0.706	0.941	0.199–4.442	0.938
Tertiary	1.256	0.310–5.086	0.749	1.086	0.241–4.895	0.914
**Race**						
African	-	-	-	-	-	-
Asian	1.591	0.318–7.952	0.572	-	-	-
Others	0.795	0.072–8.824	0.852	-	-	-
**Employment status**						
Salaried employment	-	-	-	-	-	-
Self-employment	1.382	0.925–2.063	0.114	1.362	0.883–2.102	0.163
Unemployed	1.278	0.768–2.129	0.345	1.247	0.696–2.234	0.459
**Use of hormonal method**						
Yes	2.524	1.782–3.574	< 0.0001	2.695	1.869–3.886	< 0.0001
No	-	-	-	-	-	-
**Presence of chronic illness**						
Yes	0.602	0.351–1.030	0.064	0.769	0.300–1.971	0.585
No	-	-	-	-	-	-
**Use of chronic medication**						
Yes	0.564	0.304–1.048	0.07	0.77	0.258–2.296	0.639
No	-	-	-	-	-	-
**Religion**						
Christian	-	-	-	-	-	-
Other‡	0.652	0.226–1.877	0.428	-	-	-
**Smoking status**						
Yes	1.032	0.52–2.119	0.932	-	-	-
No	-	-	-	-	-	-
**Age of participants**	0.991	0.968–1.015	0.454	1.01	0.979–1.040	0.561
**Age of partner**	0.993	0.971–1.014	0.502	-	-	-
**Alcohol use**						
Within limits	-	-	-	-	-	-
None	0.969	0.528–1.780	0.92	0.977	0.510–1.871	0.943
Excessive	0.609	0.305–1.217	0.16	0.582	0.279–1.212	0.148
**Parity**						
Nulliparous	-	-	-	-	-	-
Multiparous	0.959	0.670–1.372	0.818	-	-	-
**Miscarriage**						
No	-	-	-	-	-	-
Yes	0.539	0.286–1.018	0.057	0.613	0.311–1.211	0.159

FSD, female sexual dysfunction; CI, confidence interval; OR, odds ratio.

† , Others in ‘relationship status’ included ‘engaged’, ‘in a relationship’, ‘dating’, ‘good friends’, ‘couple’, ‘co-habiting’.

‡ , Others in religion’ included ‘atheist’.

The factor that was found to have a significant association with FSD was the use of hormonal contraception, which had an adjusted odds ratio of 2.695 (1.869–3.886, *p* < 0.0001). Other factor associations that were found not to be significant included self-employment, adjusted odds ratio of 1.362 (0.883–2.102, *p* < 0.163); unemployment, adjusted odds ratio of 1.247 (0.696–2.234, *p* < 0.459); alcohol intake (excess of recommended limits), adjusted odds ratio of 0.582 (0.279–1.212, *p* < 0.148); having a history of miscarriage(s), adjusted odds ratio of 0.613 (0.311–1.211, *p* < 0.159); having a chronic illness, adjusted odds ratio of 0.769 (0.300–1.971, *p* < 0.585); and the use of chronic medication, adjusted odds ratio of 0.77 (0.258–2.1, *p* < 0.59).

The above-named factors showed a trend towards significant association with FSD, as described in Model I of Table 4, but the study was not powered enough to show this association.

## Discussion

The present study showed that the overall prevalence of FSD in women in the study group was 38%. In comparison, the prevalence of FSD has been reported to be much higher, as high as 72.8%, in a Ghanaian prospective cross-sectional survey by Amidu et al. and 63% in a Nigerian cross-sectional study by Fajewonyomi et al. even with the study populations having had many similar sociodemographic characteristics.^7,8^

The prevalence of FSD in the women using hormonal contraception was 51.5%, compared to 29.6% among those using non-hormonal contraception, which was noted to be significantly different. On the same note, Wallwiener et al. reported that 36.7% of subjects using oral hormonal contraceptives and 31.3% of subjects using non-oral hormonal contraceptives were found to have FSD as compared to 27.4% of subjects using non-hormonal contraceptives.^9^ Although there exists contrary evidence that hormonal contraception has shown to increase positive indicators of sexual functioning as seen in a prospective study conducted by Guida et al.^10^ in Italy, and in a similar study conducted by Higgins et al.^11^ that showed more positive method-related changes in women using hormonal contraception, there is not much evidence of the effect of the scores of sexual functioning and comparator prevalence studies of FSD in the two groups described. In the present study, the use of hormonal contraception and the difference in the prevalence of FSD in the two groups were noted to be statistically significant.^10,11^

Alcohol intake was not found to have an effect on FSD in women who took alcohol within and in excess of the recommended limits. This may be attributed the fact that only 30% of the study sample took alcohol at all. In comparison to this, in a study conducted in Ghana by Amidu et al., alcohol intake was the only significant risk factor associated with FSD. This may be attributed to the physiological effects of alcohol on the female genitalia and the psychological phenomena of ‘alcohol myopia’ and ‘alcohol expectancy’. These are cognitive–physiological theories that are explained as a consequence of alcohol’s narrowing of perceptual and cognitive functioning. This can affect the expressions and emotions of women, and are associated with thoughts of pleasure, sexual performance or distress.^7^

History of miscarriage(s) was also not found to have a significant association with FSD in our study. This is contrary to findings of Shreffler et al. in 2011 who discussed how loss of pregnancy had a lasting effect of emotions of guilt or distress that had strong psychological implications, which could eventually lead to a significant effect on sexual function.^12^

There was no significant association of having a chronic illness and the use of chronic medication with FSD in our study. This is contrary to most studies that show a consistent proportional negative effect of chronic disease on sexual function. Many chronic illnesses, such as diabetes, HIV and rheumatoid arthritis, exert unwanted effects via pathophysiological mechanisms, eventually reducing sexual satisfaction scores in the women. The most common chronic illness present in our study population was hypertension. As explained above, only a small percentage (12.9%) of women in our study population had any chronic illness, had a young median age (32 years) and only 9.7% used any chronic medication; therefore, our findings are contrary to those of Doumas et al. in 2006 that showed a significant association of having hypertension with FSD in a larger study of sexually active, hypertensive women.^13^

The present study also showed that being self-employed or unemployed did not have a significant effect on sexual dysfunction. This was in comparison to the association of employment status and FSD that has been shown in many different studies conducted in different parts of the world. This is attributed to the assumption that women who are more financially stable are likely to have higher levels of confidence and self-esteem, contributing to higher sexual satisfaction scores than unemployed women. Employed women also tend to have better interpersonal skills that are associated with better desire and more willingness to have sexual activity.^14^

There was no significant effect of smoking on FSD in our study population, which is contrary to findings reported in other studies that have shown stronger associations. This is related to mechanisms of the negative effects of nicotine on the vascular system, reducing genital blood flow by inhibiting vasoactive substances such as endothelial relaxing factor and nitric oxide. This association was not seen in the present study, which is most likely because of the small number (5.8%) of active smokers in the population compared to larger populations of smokers in Europe and the United States.

The study participants were noted to be a generally young population with small age gaps with their partners. A large number were non-smokers and did not take alcohol, and was reflected as very few of them having chronic illnesses and fewer of them using any chronic medications. Majority of them shared characteristics such as being married, having a tertiary education and being employed. An expected finding was that most women belonged to the African race, were Christians and had had more than one pregnancy.^14^

It was noted in the present study that more participants were using non-hormonal methods of contraception than hormonal methods, mainly male and female condoms, and natural methods. In the group of hormonal contraception users, most were using oral contraceptives and implants. This is contrary to the report of the Kenya Demographic and Health Survey, which stated a higher prevalence of hormonal contraception with majority using injectable methods.^4^

Increasing age of participants and higher ages of their partners have shown to have a significant negative effect on sexual dysfunction as seen in other studies, but this did not reflect in our findings; however, it could be largely because of a younger mean age of participants in the present study and smaller age gaps with their partners as seen in the present study population.^14^

The present study did not find a significant association between the level of education and FSD. This association has not been clearly defined in the literature as well as with some studies showing an inverse effect of the years of education and prevalence of FSD such as the cross-sectional study conducted in Nigeria by Fajewonyomi et al. Other larger studies still report a proportionate effect on FSD. The association may not have come out very clear in the present study population as majority of the participants had attained tertiary level of education – there were very few with lower levels of education.^14^

The limitations of this study were that the findings may not be generalisable to the entire population of Kenya as it was conducted in a tertiary hospital, in the private health care clinical setting, with majority of the participants having high education levels, more financial security and health insurance, and higher employment levels. This also limited the ability of the study to elaborate on other associations that were close to significant in the study sample such as employment, education levels and religion. Also, stratification of the different types of FSD was not done as part of the data analysis in this study.

Further research to build on womens’ sexual health and sexual function should be conducted in more centres including rural settings in Kenya after adequate translation and validation of FSFI questionnaire to Kiswahili and in populations where some of the expected associations can be better evaluated and to build on this knowledge by further elaborating the association of each individual type of hormonal contraception with FSD.

## Conclusion

The present study found a high prevalence of FSD in the study population. The most common methods of contraception were the use of male and female condoms and natural methods followed by the use of oral contraceptives and implants. We noted a strong association of FSD with the use of hormonal contraception. Other factors were found to have close to significant association with FSD, but this study was not adequately powered to show this association. These factors included self-employment and unemployment, use of alcohol within and in excess of the recommended limits and a history of miscarriages; the presence of any chronic illness or use of any chronic medication showed less significant associations with FSD.
